# Plant photoreceptors and their signalling components in chloroplastic anterograde and retrograde communication

**DOI:** 10.1093/jxb/erac220

**Published:** 2022-05-30

**Authors:** Jonathan H C Griffin, Gabriela Toledo-Ortiz

**Affiliations:** Lancaster Environment Centre, Lancaster University, Lancaster, UK; Lancaster Environment Centre, Lancaster University, Lancaster, UK; University of Vienna, Austria

**Keywords:** Anterograde signals, chloroplast, cryptochrome photoreceptors, GUN mutants, HY5, MEcPP, photomorphogenesis, phytochrome photoreceptors, plastome, retrograde signals, tetrapyrroles

## Abstract

The red phytochrome and blue cryptochrome plant photoreceptors play essential roles in promoting genome-wide changes in nuclear and chloroplastic gene expression for photomorphogenesis, plastid development, and greening. While their importance in anterograde signalling has been long recognized, the molecular mechanisms involved remain under active investigation. More recently, the intertwining of the light signalling cascades with the retrograde signals for the optimization of chloroplast functions has been acknowledged. Advances in the field support the participation of phytochromes, cryptochromes, and key light-modulated transcription factors, including HY5 and the PIFs, in the regulation of chloroplastic biochemical pathways that produce retrograde signals, including the tetrapyrroles and the chloroplastic MEP-isoprenoids. Interestingly, in a feedback loop, the photoreceptors and their signalling components are targets themselves of these retrograde signals, aimed at optimizing photomorphogenesis to the status of the chloroplasts, with GUN proteins functioning at the convergence points. High light and shade are also conditions where the photoreceptors tune growth responses to chloroplast functions. Interestingly, photoreceptors and retrograde signals also converge in the modulation of dual-localized proteins (chloroplastic/nuclear) including WHIRLY and HEMERA/pTAC12, whose functions are required for the optimization of photosynthetic activities in changing environments and are proposed to act themselves as retrograde signals.

## Teaser

Phytochrome and cryptochrome photoreceptors are essential for tuning photomorphogenesis and chloroplast functions, yet their integration in the interorganellar communication cascades for proper environmental responsiveness is just beginning to be addressed.

## Introduction

### Photoreceptor activity is critical to chloroplast development and photosynthetic metabolism

Plant photoreceptors utilize light to coordinate growth, development, and photosynthetic functions in a changing environment. Mechanistically, both the red/far-red light-sensing phytochromes (phys) and the blue light-sensing cryptochromes (CRYs) are essential in the orchestration of large-scale changes in gene expression to modulate photomorphogenesis (Franklin and Quail, 2010; Yu *et al.*, 2010). Prominently, their transcriptional cascades facilitate the onset of plastid development, greening, the production of photosynthetic pigments, and the set up and maintenance of photosynthetic metabolism, among other light-controlled responses (Franklin and Quail, 2010; Yu *et al.*, 2010).

Beyond the photoreceptors’ downstream activation of thousands of nuclear genes whose protein products have a chloroplastic function including in photosynthesis (Ohgishi *et al.*, 2004; Stephenson and Terry, 2008; Chen and Chory, 2011), recent research hints at the involvement of the phy and the CRY photoreceptors in the global transcriptional, post-transcriptional, and post-translational modulation of plastid-encoded genes (Chen *et al.*, 2010; Oh and Montgomery, 2014; Facella *et al.*, 2017; Yoo *et al.*, 2019; Griffin *et al.*, 2020). Hence, the light photoreceptors not only have a central role in the anterograde (nucleus to plastid) signalling cascades, but also intertwine with the retrograde (plastids to nucleus) signals for the optimization and maintenance of plastid functions and metabolism.

### Phytochromes and cryptochromes in anterograde signalling

The anterograde signalling pathways are nuclear to chloroplast communication channels involved in setting and tuning chloroplast development and functions, circadian responses, and photosynthesis (Berry *et al.*, 2013; Atkins and Dodd, 2014; Leister and Kleine, 2016). Anterograde signals became necessary following the ancestral endosymbiotic event that originated the chloroplasts. Through evolution, many of the genes from the chloroplast genome (the plastome) were transferred to the nuclear genome (Garrido *et al.*, 2020), but remained functionally associated with the chloroplast. Also, by the acquisition of an N-terminal transit peptide, their protein products gained targeting to the chloroplasts after transcription in the nucleus and translation in the cytoplasm (Wollman, 2016).

The tight regulation of these nuclear genes coding for chloroplast-functioning proteins (globally known as photosynthesis-associated nuclear genes, *PhANG*s) is critical for chloroplast biogenesis and photosynthesis, and the photoreceptors are essential for tuning their transcriptional responses in changing light environments (Larkin and Ruckle, 2008; Pogson *et al.*, 2015; Calderon and Strand, 2021). Both the phys and the CRYs regulate the global light responsiveness of the *PhANG*s through the activation or repression of multiple transcription factors including: the bZIP-LONG HYPOCOTYL 5 (HY5) (Osterlund *et al.*, 2000; Toledo-Ortiz *et al.*, 2014); the basic helix–loop–helix phytochrome-interacting factors (PIFs) (Franklin and Quail, 2010; Leivar and Quail, 2011); and the GARP proteins GOLDEN2-LIKE 1 and 2 (GLK1 and GLK2) (Waters *et al.*, 2009; Leister and Kleine, 2016).

HY5 is a master transcription factor in the control of photomorphogenic responses (Gangappa and Botto, 2016) capable of integrating red phy and blue CRY responses. Both photoreceptors tune HY5 abundance in the nucleus by down-regulating the COP1-dependent ubiquitination of HY5 and allowing its accumulation in the light (Osterlund *et al.*, 2000). HY5 binds to the promoters of nearly 4000 genes and controls a wide range of developmental processes including the activation of photosynthesis-associated genes (J. Lee *et al.*, 2007; Gangappa and Botto, 2016), photopigment and antioxidant accumulation (J. Lee *et al.*, 2007; Shin *et al.*, 2007; Toledo-Ortiz *et al.*, 2014), as well as circadian and growth responses (J. Lee *et al.*, 2007; Hajdu *et al.*, 2018).

The PIFs are negative modulators of photomorphogenesis that are degraded in the light after the activation of phys, and are involved in promoting skotomorphogenesis and shade avoidance responses (Leivar and Quail, 2011; Yoo *et al.*, 2019). While their turnover and stability are principally regulated by the phys, CRYs can repress the transcription of *PIF4* without affecting its protein stability (Ma *et al.*, 2016), and may also protect PIF5 from phy-mediated degradation in low blue light conditions (Pedmale *et al.*, 2016). PIFs promote skotomorphogenesis (Wang *et al.*, 2022) including the down-regulation of genes involved in photopigment biosynthesis (Shin *et al.*, 2007; Stephenson *et al.*, 2009) and chloroplast development and function (Leivar and Monte, 2014).

The GLK transcription factors target genes involved in light harvesting and chlorophyll biosynthesis through direct binding to their light-sensitive promoters, and are required for chloroplast development (Waters *et al.*, 2009). In addition, *GLK1* and *GLK2* transcript accumulation is dependent on red phys and blue light, and the *glk1 glk2* double mutant has reduced accumulation of transcripts for photosynthetic genes and lower chlorophyll content when grown in blue light (Waters *et al.*, 2009), hinting at their involvement with CRY signalling cascades leading to greening.

Beyond the important role of the CRYs and phys in the transcriptional response of chloroplast functioning genes, recent research provides evidence that the phys are also key regulators of ribosome biogenesis and translation during late leaf development, with a global modulation of mRNAs that code for components of aminoacyl-tRNA biosynthesis, elongation factors, and ribosomal subunits (Romanowski *et al.*, 2021). Active phyB has also been reported to interact with cytosolic RNA-binding proteins, including PENTA1 (PTN1), to inhibit the translation of mRNAs for genes such as protochlorophyllide (PORA) involved in chlorophyll biosynthesis (Paik *et al.*, 2012).

In addition, beyond the activation of the nuclear genome for the production of the chloroplastic proteins encoded by it, chloroplast functions require coordination of gene expression with the plastome, wherein essential subunits of the photosynthetic complexes are encoded. As such, part of the anterograde signalling pathways relates to the delivery of information for tuning the chloroplast genome in response to the environment (Oh and Montgomery, 2014; Facella *et al.*, 2017; Griffin *et al.*, 2020). CRY2 overexpression studies in tomato defined a broad contribution to the plastome expression in long days (58% of the 114 plastome ORFs), with an up-regulation of PSII (*psb*), PSI (*psa*), and Cyt *b*_6_*f* (*pet*) transcripts and down-regulation of multiple large and small ribosomal proteins (*rps* and *rpl*). In addition, genes coding for other photosynthetic complexes such as NADH dehydrogenase (*ndh*) and ATP synthase (*atp*) showed a mixed regulation (Facella *et al.*, 2017). A similar analysis in Arabidopsis for the *phyB* mutant in short days revealed an analogous capacity to globally regulate the transcripts of 55 out of 80 plastome-encoded genes (Michael *et al.*, 2008; Griffin *et al.*, 2020). While in most cases phyB function was related to transcript up-regulation, down-regulation of key *atp*, *ndh*, *psa*, and *psb* transcripts was also detected (Griffin *et al.*, 2020).

Alongside these reports, bioinformatic studies of genomic datasets for Arabidopsis *cry1 cry2* and *phyabcde* revealed a significant contribution of red phys and blue CRYs to the light-dependent expression of nuclear-encoded genes whose protein products are linked to the transcriptional, post-transcriptional, and translational control of the plastome (Griffin *et al.*, 2020). Among the light-modulated gene families identified were the sigma factor transcriptional cofactors required for the activity of PLASTID-ENCODED POLYMERASE (PEP) (Oh and Montgomery, 2014; Börner *et al.*, 2015); the pentatricopeptide domain-containing (PPR) and the tetratricopeptide domain-containing (TPR) families of RNA-binding proteins with a role in the plastome post-transcriptional events (Lamb *et al.*, 1995; Ruwe *et al.*, 2011). In addition, for the blue CRYs, genes coding for RNA-recognition motif (RRM) RNA-binding proteins with an annotated role in post-transcription and for tRNA ligase and large ribosomal protein (RPL) related to translation were identified (Griffin *et al.*, 2020). In this context, HY5 was singled out as a relevant transcription factor delivering light cues to the ‘plastome-regulatory gene network’. Gene targets include the sigma factors and the PLASMID TRANSCRIPTIONALLY ACTIVE CHROMOSOME class (pTACs), involved in plastome transcription, and the PPR and the TPR proteins probably involved in post-transcriptional processes.

These early studies provide evidence that the photoreceptors and their signalling components are central in the anterograde signalling cascades to tune the global expression of the plastome in response to environmental signals, but the detailed mechanistic insights remain to be understood.

### The chloroplast retrograde signalling pathways

Retrograde signalling pathways are a second type of interorganellar communication channels used by the plastids to relay information to the nucleus in response to a range of stresses or external stimuli for the optimization of growth and for shaping photosynthetic and chloroplast biogenic responses (Kusnetsov *et al.*, 1996; Leister and Kleine, 2016; Hernández-Verdeja and Strand, 2018). Retrograde signalling during chloroplast biogenesis (defined as the transition from etioplasts or proplastids to chloroplasts), germination, or early seedling development, is referred to as biogenic signalling (Pogson *et al.*, 2008). Biogenic signalling tunes-up and -down hundreds of nuclear-encoded genes whose protein products function in the chloroplast (Chan *et al.*, 2016). A variety of intermediates from chloroplastic metabolic pathways, including tetrapyrroles, methylerythritol phosphate (MEP) pathway isoprenoids, phosphoadenosines, carbohydrates, carotenoid oxidation products, and reactive oxygen species (ROS), have been identified as biogenic signals emitted by the chloroplast to deliver information to the nucleus. The biogenic retrograde signalling pathways have been recently reviewed in detail (Chi *et al.*, 2015; Terry and Bampton, 2019).

The crucial contribution of retrograde signalling to seedling survival has been assessed in mutants with impaired retrograde signalling capabilities, and through pharmacological approaches that induce stress in the chloroplasts (Pogson *et al.*, 2008; Chan *et al.*, 2016). Common retrograde signal activators include lincomycin (an inhibitor of plastid translation that blocks plastid development) and norflurazon (an inhibitor of carotenoid biosynthesis that induces photobleached chloroplasts). These chemical agents trigger a reduction in the expression *PhANG*s, including those coding for light-harvesting complex B (*LHCB*) proteins and the Rubisco small subunit (*RBCS*), that are common marker genes for assessing retrograde signal activity (Susek *et al.*, 1993; Ruckle *et al.*, 2012). In Arabidopsis, forward mutagenic screens coupled with the use of norflurazon identified the *gun1* (genome uncoupled) mutants with altered accumulation of *PhANG*s, such as *CAB* (Chl *a*/*b*-binding protein) (Susek and Chory, 1992; Susek *et al.*, 1993; Mochizuki *et al.*, 2001).

A second type of retrograde signalling involves operational signals that occur after chloroplast biogenesis and in response to stress conditions to induce adjustments in chloroplast homeostasis (Pogson *et al.*, 2008; Chan *et al.*, 2016). Examples of identified operational signalling pathways include the regulation of PSII overexcitation via β-cyclocitral (Ramel *et al.*, 2012), and the methylerythritol cyclodiphosphate (MEcPP) pathway (Jiang and Dehesh, 2021).

This review focuses on the involvement of the photoreceptors in the regulation of the biogenic and operational pathways, including links to the GUN signalling pathways and MEcPP pathway, and novel insights on dual-localized proteins in chloroplast to nuclear signalling (Qin *et al.*, 2010; Martín *et al.*, 2016; Ren *et al.*, 2017; Jiang and Dehesh, 2021).

### The intertwining of retrograde signalling and photoreceptor-dependent pathways

While connections between plastid retrograde signalling and light signalling have been made for decades, most of the mechanisms involved remain elusive (Kusnetsov *et al.*, 1996; Larkin and Ruckle, 2008; Xu *et al.*, 2016). In 1996, Kusnetsov *et al*. examined the effect of the overlap between plastid-derived retrograde signals and light-derived signals on functional *PhANG* promoter sequences. These authors provided early evidence that chloroplast-derived retrograde signals and light signalling pathways act on the same *cis*-acting elements (such as L-, I-, and G-boxes), and could regulate the same processes, suggesting an intertwining of the pathways. Since then, G-boxes have been characterized as important light-responsive elements (LREs) bound by multiple phy and CRY downstream signalling components including HY5 and the PIFs (Chattopadhyay *et al.*, 1998; Leivar and Quail, 2011).

Experimental evidence also supports that the activation of retrograde signalling pathways by lincomycin and norflurazon represses or delays plant photoreceptors’ promotion of photomorphogenesis, including chloroplast biogenesis and greening processes (Susek *et al.*, 1993; Ruckle *et al.*, 2012). There is also a clear overlap between the gene targets of the biogenic retrograde signalling pathways and the photomorphogenic cascades initiated by the phys and the CRYs (Ohgishi *et al.*, 2004; Tepperman *et al.*, 2006; Ruckle *et al.*, 2012; Zhao *et al.*, 2019). Examples of common targets include the subunits of *LHCB* and *RBCS* (Reed *et al.*, 1994; Mazzella *et al.*, 2001; Vinti *et al.*, 2005; Woodson *et al.*, 2011). Furthermore, RNA-seq experiments with norflurazon have provided evidence that the genes coding for phyA and for light-modulated transcription factors such as HY5 are up-regulated, and *PIF4* and *PIF7* are down-regulated upon activation of retrograde signal pathways (Zhao *et al.*, 2019), giving support to the hypothesis that photoreceptors and their signalling components and retrograde signals highly intersect and do not operate independently of each other.

Beyond the chemical activators of retrograde signals, high light (HL) is also an important trigger (Szechyńska-Hebda and Karpiński, 2013), and photoreceptors are part of the perception and responsiveness to HL (Kreslavski *et al.*, 2020). ROS including hydrogen peroxide (H_2_O_2_), superoxide anions (O^2–^), and singlet oxygen (^1^O_2_) are chemical derivatives of O_2_ produced by metabolic processes in plants (Apel and Hirt, 2004). In HL irradiances, chloroplasts increase H_2_O_2_ production by PSI and ^1^O_2_ production by PSII (Kanervo *et al.*, 2005; Krieger-Liszkay, 2005). While H_2_O_2_ has been shown to move out of isolated chloroplasts *in vitro*, providing it with the capacity to act as an initiator of retrograde signalling (Mubarakshina *et al.*, 2010), ^1^O_2_ cannot leave the chloroplast due to its short half-life (Gorman and Rodgers, 1992), and therefore secondary messengers yet to be identified must be involved in the transmission of the ^1^O_2_ signal to the nucleus.

In addition to ROS, HL stress also generates 12-oxophytodienoic acid (OPDA) and oxylipin-derived retrograde signals (Gollan and Aro, 2020). Among the targets of these retrograde signalling cascades is EARLY LIGHT INDUCIBLE PROTEIN1 (ELIP1) (Gollan and Aro, 2020), a thylakoid protein induced during de-etiolation and in response to HL stress (Rossini *et al.*, 2006). ELIPs may participate in enhancing the photoprotective capacity of the plant (Casazza *et al.*, 2005; Rossini *et al.*, 2006) and, under HL, CRY1 and HY5 modulate the induction of *ELIP1* (Kleine *et al.*, 2007). As part of these cascades, a second cross-regulatory point is the modulation of heat shock protein (HSP) chaperones (including HSP90) which are HY5 targets and participate in the tetrapyrrole-mediated plastid signalling to repress *PhANG*s under oxidative stress (Kindgren *et al.*, 2012).

These examples illustrate that photoreceptor activity is crucial for the set up of the protective responses against HL stress, as well as for the communication channels activated by high irradiances. Likewise, phys and CRYs promote the activation of nuclear genes for the biosynthesis of carotenoids and anthocyanins to deal with excess light (Kreslavski *et al.*, 2020). Accordingly, the *cry1phyAB1* and *phyAB1B2* mutants in *Solanum lycopersicum* present additive HL stress phenotypes, including reductions in photopigment content and photosynthetic activity, and lower transcript accumulation of photosynthesis-associated genes encoded in both the plastome and the nuclear genome (*PhANG*s and *PhAPG*s) (Kreslavski *et al.*, 2020). Furthermore, the more acute HL damage observed for *cry1phyAB1* may point to a larger contribution of CRY1 to HL tolerance and responsive mechanisms in tomato plants.

Studies in Arabidopsis further support this primary role of CRY1 in managing photoprotective and HL responses, and single out HY5, whose transcript and protein accumulate in HL, as one of the light signalling components involved (Kleine *et al.*, 2007). In addition to a HL-sensitive phenotype including the photo-inactivation of PSII, the *cry1* mutant exhibits at a transcriptomic level misregulation of 77 HL-induced genes, with 26 of them also misregulated in *hy5* (Kleine *et al.*, 2007). Interestingly, a further 39 genes showed altered patterns of accumulation in *hy5*, but not in *cry1*, indicating that HY5 participates in both HL–CRY1-dependent and HL-responsive but CRY-independent pathways.

Additional evidence from studies in emerging rice seedlings grown under high blue or high red light and lincomycin supports both an integration and a differential contribution of light quality and photoreceptor activity to seedling photomorphogenesis and non-photochemical quenching mechanisms to tolerate the excess light (Duan *et al.*, 2020). In this context, in high red light conditions, retrograde signal activators induced photobleaching, but in high blue light, enhanced carotenoid and chlorophyll production contributed to a stronger HL stress tolerance, in a mechanism likely to be dependent on CRYs (Kleine *et al.*, 2007; Duan *et al.*, 2020; Richter *et al.*, 2020).

In summary, HL responses involve both photoreceptors (CRYs and phys) and light signalling components (such as HY5) capable of sensing and responding to both HL and retrograde signals to tune growth and development with the status of the chloroplast. Current studies also support the conservation of these HL-induced retrograde signalling cascades between monocot and dicot plants (Duan *et al.*, 2020).

### Photoreceptors, HY5, and GUN1 in the convergence of photomorphogenesis and retrograde signalling

The *GUN* genes (*GUN1–GUN6*) were identified in the ‘gun mutant screens’ using norflurazon to activate retrograde signals (Susek and Chory, 1992; Susek *et al.*, 1993; Mochizuki *et al.*, 2001; Woodson *et al.*, 2011). GUN2–GUN6 play roles in the tetrapyrrole biosynthesis pathway and, while the full functional role of GUN1 remains to be addressed, experimental evidence also supports GUN1 modulation of tetrapyrroles by direct binding to both haem and porphyrins (Shimizu *et al.*, 2019).

Tetrapyrroles, as either bilins or porphyrins, have important functions in multiple biological processes, including respiration and photosynthesis, and are active in light absorption, electron transfer, and oxygen binding (Shimizu *et al.*, 2019). Tetrapyrrole biosynthesis takes place in the plastids and involves two key pathways branching from protoporphyrin IX: the chlorophyll branch, ending in production of Chls *a* and *b*; and the haem branch, ending in phytochromobilin (the chromophore used by the red and far-red light phy photoreceptors) (Bae and Choi, 2008; Li *et al.*, 2011). A tight regulation of tetrapyrrole biosynthesis is required to avoid cellular damage by the generation of ROS.

As the *gun* mutants involve mutations within the tetrapyrrole biosynthetic pathway, the metabolites therein are considered key retrograde signals for chloroplast development (Leister and Kleine, 2016). In the chlorophyll branch of the tetrapyrrole biosynthesis pathway, *GUN5* encodes a gene for the H subunit of magnesium chelatase (MgCh), involved in the transition between protoporphyrin IX (ProtoIX) and magnesium protoporphyrin IX (Mg-ProtoIX) (Mochizuki *et al.*, 2001). *GUN4* encodes an activator of MgCh that also contributes to the accumulation of Mg-ProtoIX (Larkin *et al.*, 2003). Mg-ProtoIX has been proposed as a one of the important signalling molecules for retrograde signalling (Kindgren *et al.*, 2011), linked to the reduction in transcript levels of *PhANG*s, including *LHCB* and *RBCS* (Shimizu *et al.*, 2019). However, beyond *gun4* and *gun5*, other mutants for genes encoding subunits for the Mg-ProtoIX complex do not display a *gun* phenotype, making the role of this metabolite in retrograde signalling unclear at present (Mochizuki *et al.*, 2001; Wu and Bock, 2021).

The haem branch of tetrapyrrole synthesis is initiated by GUN6 (also known as plastid FERROCHELATASE 1, FC1) that converts ProtoIX to protohaem by inserting Fe^2+^. Protohaem is converted first to biliverdin IX by GUN2 (encoding haem oxygenase), and finally to 3Z-phytochromobilin by GUN3 (phytochromobilin synthase). Evidence that haem may function as a second type of retrograde signalling molecule has been provided by the characterization of *gun6-1D*, a dominant mutant allele overexpressing *FC1*, and promoting the flow of tetrapyrroles into the haem branch, with consequent up-regulation of *PhANG* transcripts (Woodson *et al*., 2011).

While the specific mechanisms through which photoreceptor signalling pathways are involved in the generation, regulation, and response to GUN retrograde signals have yet to be fully elucidated, tetrapyrrole biosynthesis is induced by light, as previously reviewed (Kobayashi and Masuda, 2016), with the contribution of light signalling transcription factors including HY5 (K.P. Lee *et al.*, 2007; Kobayashi *et al.*, 2012a, b), the PIFs (Shin *et al.*, 2009; Leivar and Quail, 2011), and GLK1 and GLK2 (Waters *et al.*, 2009).

In particular, *GUN1* is a gene of high interest as an integratory point for light and retrograde signalling pathways. *GUN1* encodes a chloroplast-localized protein containing a PPR (Koussevitzky *et al.*, 2007). PPRs are known post-transcriptional regulators of plastid gene expression (Ruwe *et al.*, 2011), but the functional role of GUN1 protein is still under exploration. Of all *gun* mutants, *gun1* exhibits the strongest de-repression of *PhANG* expression in lincomycin (Koussevitzky *et al.*, 2007), and *GUN1* transcript accumulation is light responsive and dependent on the phys in red light (Hu *et al.*, 2013). During de-etiolation, GUN1 is active and involved in cotyledon expansion and hypocotyl elongation (Ruckle *et al.*, 2007; Ruckle and Larkin, 2009), with *gun1* also displaying a delayed greening phenotype. As such, GUN1 probably represents a crosstalk point between the photoreceptor signalling cascades and the plastid signals that tune chloroplast greening and growth responses (Mochizuki *et al.*, 1996; Ruckle *et al.*, 2007; Pesaresi and Kim, 2019; Wu *et al.*, 2019; Wu and Bock, 2021).

Further support for this possibility has been provided by additional *gun* genetic screens, where an allele of *cry1* that shares similar phenotypes with *gun1-1*, including defects in plastid to nucleus signalling affecting *LHCB* and *RBCS* transcript accumulation, was identified (Ruckle *et al.*, 2007). Double mutant analysis of *gun1-101 cry1* grown in HL showed an additive phenotype for their effects on *LHCB* accumulation and deficiencies in chlorophyll accumulation, indicating that GUN1 and CRY1 may be partially redundant in modulating *LHCB* via parallel pathways that converge. A similar phenotype of defective *LHCB* accumulation was observed for the *gun1-101 hy5* double mutant, suggesting that this CRY1-dependent pathway requires HY5. Likewise, *phyB gun1-1* double mutants accumulated more *LHCB* than *gun1-1* single mutants when treated with lincomycin, providing evidence that phyB may also be a *gun* mutant, contributing to the repression of *LHCB*, but only when GUN1 is inactive (Ruckle *et al.*, 2007).

In summary, the light/photoreceptor-dependent modulation of *GUN1*, together with the additive phenotypes between *gun1* and photoreceptor mutants, point at signal integration between the light cascades and the retrograde signals via GUN1, with HY5 as a potential ‘convergence of signals point’ for which full mechanistic insights await full dissection.

### Phytochrome-dependent GLK tuning of PhANGs is antagonized by GUN signalling

An additional molecular link identified between the GUN pathways and the photoreceptor signalling cascades during de-etiolation was recently uncovered (Martín *et al.*, 2016). These authors showed that during de-etiolation, the phy photomorphogenic signals and the GUN1 biogenic retrograde signalling pathways converge to antagonistically control photomorphogenesis. Notably, Arabidopsis plants grown in red or white light, with inhibition of chloroplast biogenesis induced by lincomycin or norflurazon, showed elongated hypocotyls and unexpanded cotyledons lacking chlorophyll—phenotypes associated with dark-grown seedlings. These observations give support to a retrograde signal-dependent tuning down of light-dependent pathways with suppression of photomorphogenic development.

Interestingly, genomic studies showed that >343 photomorphogenesis-associated genes involved in de-etiolation and greening are co-repressed both by lincomycin-induced/GUN1-derived retrograde signals and by the PIFs in the dark. This transcriptional effect was further supported by the characterization of the *pifq* (*pif1 pif3 pif4 pif5*) mutant, for which treatment with lincomycin restored the transcriptomic profile of PIF-repressed genes to wild-type levels, indicating a parallel pathway to GUN1 in response to chloroplast dysfunction (Martín *et al.*, 2016). An analysis of the DNA-binding motifs in the promoters of the genes co-repressed by both lincomycin and PIFs identified an enrichment in GLK-binding motifs (Martín *et al.*, 2016). *GLK1* encodes a transcription factor that is both phy/light induced and PIF repressed, and whose down-regulation by retrograde signals in a GUN1/GUN5-dependent manner is reported (Kakizaki *et al.*, 2009; Waters *et al.*, 2009). In addition, characterization of overexpressing lines for *GLK1* and *GLK2* placed them as *gun* mutants themselves (Leister and Kleine, 2016). As part of the GUN1/GLK1-mediated responses, the B-box gene *BBX16* has been identified as a directly induced target of GLK1 for the promotion of photomorphogenesis, and whose transcription is repressed in a GUN1/GLK1-dependent manner upon chloroplast damage, as well as in response to norflurazon treatment (Zhao *et al.*, 2019; Veciana *et al.*, 2022).

Along with the links between retrograde signalling and GUN signalling in the light, evidence also suggests that these pathways may operate in darkness, with the involvement of COP1 and the PIFs. Support for this possibility comes from experiments on etiolated Arabidopsis *pifq* seedlings that, when grown in the presence of lincomycin, show a restoration to phenotypes present in wild-type etiolated seedlings, including suppression of cotyledon separation and sustainment of apical hook curvature and of appressed cotyledons (Martín *et al.*, 2016). In addition, lincomycin also reduces the transcript accumulation of photomorphogenesis-associated genes such as *LHCB1* in dark-grown *cop1*, and of 354 transcripts in dark-grown *pifq* mutants (Sullivan and Gray, 1999; Martín *et al.*, 2016). Also, recent studies of dark-grown etioplasts and pro-plastids revealed the presence of GUN1 protein in the dark, and transcriptomic studies on dark-grown wild type and *gun1-102* indicate that GUN1-mediated signals regulate nuclear gene expression in the dark with up to 4425 genes, including subunits of PSI (*PSA*) and *LHCB*, differentially expressed in dark-grown *gun1-101* compared with the wild type. These results support a significant role for GUN1 in tuning the expression in the dark of genes involved in the build-up of the photosynthetic apparatus (Hernandez-Verdeja *et al.*, 2022).

Therefore, while the molecular connections between the GUN1 retrograde signalling and the phy cascades are only beginning to be addressed, progress in the area points to retrograde signals acting as an antagonistic pathway to suppress phy-induced photomorphogenesis. In this context, GUN1 can integrate retrograde signals downstream of COP1 to tune the initiation of photomorphogenesis, including those that modulate the transcriptional responses of transcription factors required for de-etiolation and for chloroplast development such as GLK1, HY5, PIF1, PIF4, PIF5, and PIF8 (Hernández-Verdeja *et al.*, 2022).

### Photoreceptors and the MEcPP retrograde signalling pathway

Along with their roles in initiating greening and tetrapyrrole biosynthesis, phys are downstream targets of MEcPP, an isoprenoid derivative of the chloroplastic MEP pathway, and a powerful operational retrograde signalling molecule (de Souza *et al.*, 2017; Jiang *et al.*, 2019; Jiang and Dehesh, 2021) for the expression of nuclear genes involved in stress responses in plastids (Xiao *et al.*, 2012; de Souza *et al.*, 2017). The plastidial accumulation of MEcPP is induced in response to oxidative stress, HL, wounding, high temperature, and heavy metals in plants and eubacteria (Xiao *et al.*, 2012; Wang *et al.*, 2017).

A genetic screen in Arabidopsis to identify genes involved in the regulation of HYDROPEROXIDE LYASE (HPL), a stress-inducible protein in the oxylipin pathway, identified the *constitutively expressing HPL* (*ceh1*) mutant (Xiao *et al.*, 2012). *ceh1* has a mutation in HMBPP synthase (HDS) that catalyses the conversion of MEcPP to HMBPP (Ostrovsky *et al.*, 1998; Rodríguez-Concepción, 2006; Xiao *et al.*, 2012), and displays short hypocotyls in the light (Jiang *et al.*, 2019, 2020). This phenotype is caused by higher phyB protein levels induced by the overaccumulation of MEcPP (Jiang *et al.*, 2020). Higher phyB levels lead to the repression of PIF4 and PIF5 activity and to an altered accumulation of ethylene and auxin biosynthetic genes such as *ACS4*, *5*, *8*, and *YUC8* (Jiang *et al.*, 2019, 2020). Interestingly, the short hypocotyl phenotype of *ceh1* mutants was also present in seedlings grown under blue light, supporting the possibility that blue light-sensing CRYs are also linked to MEcPP accumulation and signalling (Jiang *et al.*, 2019).

While phyB is a downstream target of a MEcPP retrograde signal, phyB and transcription factors acting downstream of phyB are also critical regulators of multiple MEP pathway genes [e.g. *DXP SYNTHASE* (*DXS*), *DXP REDUCTOISOMERASE* (*DXR*), and *HMBPP REDUCTASE* (*HDR*)] from which MEcPP is derived (Chenge-Espinosa *et al.*, 2018). In particular, red light signals from phys and HY5, antagonistically transduced by PIFs, are involved in the transcriptional control of *DXS* and *DXR*, the genes in the MEP pathway that are considered rate-limiting steps and flux-controlling points (Wright *et al.*, 2014; Chenge-Espinosa *et al.*, 2018).

Together, these findings support a cross-regulation between the photoreceptors and the MEcPP retrograde signalling pathways, with phyB as both a key target of retrograde signals in red light and a regulator of their generation, in a feedback loop that adjusts photomorphogenic responses to the status of the chloroplast.

### HY5 emergence as an important integratory factor for light and multiple retrograde signalling pathways

HY5 is a master modulator of plant photomorphogenesis, including the control of de-etiolation, photopigment accumulation, hormonal levels, anthocyanin production, and tuning of reactive oxygen stress responses (Kobayashi *et al.*, 2012b; Toledo-Ortiz *et al.*, 2014; Gangappa and Botto, 2016). In the light, several pieces of evidence support the signal integratory capacity of CRY and phys signal via HY5 with retrograde signalling (Ruckle *et al.*, 2007; Kindgren *et al.*, 2012; Richter *et al.*, 2020). As such, *HY5* transcript accumulation increases in response to retrograde signal activators (Zhao *et al.*, 2019), and HY5 has been proposed to alternate between an activator and a repressor of nuclear-encoded gene expression in response to plastid dysfunction (K.P. Lee *et al.*, 2007; Ruckle *et al.*, 2007; Ruckle and Larkin, 2009).

In addition, HY5 mediates the GUN1-triggered rapid light-dependent inhibition of *PhANG*s, induced by singlet oxygen retrograde signals derived from the photo-excitation of Mg-porphyrins and the accumulation of the chlorophyll intermediate Mg-ProtoIX (Strand *et al.*, 2003; Kindgren *et al.*, 2012; Richter *et al.*, 2020). Mg-ProtoIX interaction with cytosolic HSP90 proteins leads to the repression or inactivation of nuclear-encoded *PhANG*s in a HY5-dependent manner (Kindgren *et al.*, 2012). In this pathway, GUN5–HSP90.2–HY5 is emerging as a convergence point for light and retrograde signalling cascades for the modulation of *PhANG*s. HY5 may also form—together with GUN1 and HSP90.1 (Wu *et al.*, 2019; Wu and Bock, 2021)—a second integratory node for light retrograde signals, whose full biological significance remains to be investigated.

Additionally, together with CRYs, HY5 also participates in the coordination of light and retrograde signals for anthocyanin and flavonoid accumulation (Shin *et al.*, 2007; Zhang *et al.*, 2016; Richter *et al.*, 2020). In this respect, current evidence shows that in norflurazon-treated Arabidopsis plants, GUN1/GUN5 retrograde signals can tune down the transcript accumulation of flavonoid/anthocyanin biosynthesis (*FAB*) genes, including *LEUCOANTHOCYANIDIN DIOXYGENASE* (*LDOX*), a gene whose activation depends on CRY1 and HY5 (Richter *et al.*, 2020).

As such, current studies support the participation of CRY1 and HY5 in abiotic stress-triggered retrograde signalling cascades necessary for enabling chloroplast stress responsiveness, the modulation of photoprotective pigment accumulation, and repression of the expression of the *PhANG*s.

Another reported link between HY5 and the tetrapyrrole biosynthesis-derived retrograde signalling cascades involves the sigma factors. The sigma transcriptional cofactors are nuclear-encoded genes required for the activity of the PEP (Berry *et al.*, 2013; Börner *et al.*, 2015). In Arabidopsis, there are six members of the sigma factor family, with five of them (*SIG1*, *2*, *3*, *5*, and *6*) showing red phy-, blue CRY-, or red/blue HY5-dependent transcript accumulation (Oh and Montgomery, 2013; Griffin *et al.*, 2020). For SIG2 and SIG5, links to retrograde signalling are emerging (Woodson *et al.*, 2013; Oh *et al.*, 2018) with SIG2 modulation of the expression of tRNA-Glu, an early step in tetrapyrrole, biosynthesis (Woodson *et al.*, 2013), and a reduced accumulation of *PhANG* transcripts (including *RBCS* and *LHCB* genes) in *sig2*, a phenotype that is alleviated by haem feeding. Transcriptomic studies for SIG2 have also identified under red light >2000 nuclear-encoded misregulated genes, some with roles in growth, hormonal crosstalk, stress responses, and photosynthesis (Oh *et al.*, 2018). The enrichment in *sig2* of misregulated chloroplastic/red light-responsive genes that are targets of retrograde signals supports an intersection of both pathways for the modulation, in particular, of chloroplastic acting genes and of genes active during in photomorphogenesis.

A second sigma factor, SIG5, is a light quality- and HL-responsive gene that is sensitive to DCMU-dependent retrograde signals (Mellenthin *et al.*, 2014). *SIG5* transcript accumulation is induced by CRY1 in blue light and is phy dependent in red light, with HY5 contributing to its transcriptional response in both light qualities (Mellenthin *et al.*, 2014; Griffin *et al.*, 2020). Following DCMU activation of retrograde signals derived from the inhibition of electron flow in PSII (Metz *et al.*, 1986; Mellenthin *et al.*, 2014), the accumulation of *SIG5* is down-regulated. These early studies indicate the capacity of SIG5 to integrate inputs from light and retrograde signals; however, the mechanistic insights into signal integration and biological outputs remain to be investigated. Yet, SIG2 and SIG5 as HY5- and retrograde signal-sensitive genes, have a good potential to be part of the anterograde and retrograde pathways to tune the plastid genome and the *PhANG* transcriptional responses with the blue and red photoreceptor light signals.

### HY5 and phyB in the shade-induced retrograde signalling pathways

In addition, HY5’s involvement in retrograde signals to avoid shade and optimize photosynthetic performance has been reported (Roig-Villanova *et al.*, 2007; Cagnola *et al.*, 2012; Bou-Torrent *et al.*, 2015; Ortiz-Alcaide *et al.*, 2019). In this context, HY5 is reported to respond to retrograde signals derived from functional chloroplasts to tune hypocotyl elongation, in a manner similar to its induction by phyA in low red:far red conditions to suppress elongation (Bou-Torrent *et al.*, 2015; Ortiz-Alcaide *et al.*, 2019). On the other hand, under shade, signals derived from challenged chloroplasts to de-activate phyB stimulate the activity of the PIFs to promote hypocotyl elongation and avoid shade (Ortiz-Alcaide *et al.*, 2019).

Studies using norflurazon or lincomycin treatments indicate a higher transcript accumulation of *HY5*, and HY5 protein can be detected in white- and in far-red light-enriched environments simulating canopies, but only when retrograde signals derived from functional chloroplasts are active (Ortiz-Alcaide *et al.*, 2019). Interestingly, in the absence of functional chloroplasts, phyB inactivation in response to far-red treatments is delayed, with the consequent reduction in the transcripts of shade-induced genes involved in elongation (Roig-Villanova *et al.*, 2007; Ortiz-Alcaide *et al.*, 2019).

In summary, current studies indicate antagonistic effects of phyB/PIFs and phyA/HY5 for the proper modulation of elongation responses upon impending competition. Yet, in this setting, chloroplast retrograde signals are also critical for the tuning of light quality/shade perception to the status of the chloroplast.

### Photoreceptors regulate retrograde signalling-dependent dual-localized proteins

Likewise, there is also evidence to support the involvement of the photoreceptors in the regulation of multiple dual-localized proteins that can communicate information between the nucleus and the chloroplast to tune chloroplast needs and photomorphogenic responses. WHIRLY1 (WHY1) is among such dual-localized proteins with potential to act as a retrograde signal based on a functional role in chloroplast biogenesis and a capability for translocation from the chloroplast back to the nucleus (Isemer *et al.*, 2012).

WHIRLY proteins are a small family of three genes in Arabidopsis, coding for ssDNA-binding proteins (Desveaux *et al.*, 2002; Krause *et al.*, 2005). WHY1 and WHY3 are targeted to chloroplast, and WHY2 localizes to the mitochondria (Krause *et al.*, 2005). WHY1 is involved in the transcriptional modulation of plastid-encoded and nuclear-encoded genes (Desveaux *et al.*, 2002, 2005; Isemer *et al.*, 2012). In the chloroplast, WHY1 forms part of the pTAC complexes involved in plastome transcription, and in the nucleus WHY1 stimulates the expression of pathogen response genes by an unknown mechanism (Isemer *et al.*, 2012).

The role of WHY1 as a retrograde signal occurs in response to redox changes in the thylakoid electron transport chain (Foyer *et al.*, 2014). WHY1’s alternative subcellular localization depends on light via the phyA-dependent regulation of the Calcineurin B-Like-Interacting Protein Kinase14 gene (*CIPK14)* (Qin *et al.*, 2010), coding for a protein that phosphorylates and modifies WHY1 binding affinity for different promoters (Ren *et al.*, 2017). Interestingly, *CIPK14* transcript accumulation is dependent on multiple light inputs, including transient activation by far-red light and time-dependent modulation by blue and red light (Qin *et al.*, 2010). At present, only the response to far-red light and the dependence on phyA have been investigated, but based on current studies it can be hypothesized that this phyA–CIPK14–WHY1 regulatory module may be important for the far red blocking of the greening response. It remains to be established if the observed red light induction of *CIPK14* is phyB dependent, but the blue light induction of *CIPK14* is not dependent on CRY1 CRY2 (Qin *et al.*, 2010).

A second example of the involvement of photoreceptors in the control of nucleo-chloroplastic dual-localized proteins include pTAC12/HEMERA (HMR), a member of the pTAC family that regulates PEP (Pfalz *et al.*, 2006; Chen *et al.*, 2010). *HMR* transcript accumulation is light responsive and dependent on the phys in red light and on the CRYs in blue light (Griffin *et al.*, 2020). In the nucleus, HMR acts as a transcriptional co-activator to regulate light-responsive genes, while in the plastids it associates with the PEP to induce plastid-encoded gene expression (Pfalz *et al.*, 2015; Qiu *et al.*, 2015). HMR first localizes to the plastids, like WHY1 (Grabowski *et al.*, 2008; Isemer *et al.*, 2012), and its relocation to the nucleus is proposed as part of the activation of the retrograde signal cascades (Yoo *et al.*, 2020). Currently this possibility, including the potential crosstalk with photoreceptor signalling mechanisms, remains to be fully investigated.

In summary, research supports the involvement of phys in the modulation of the activity of nuclear–chloroplastic proteins that directly or indirectly impact on the expression of the nuclear and plastid genomes. At present, only the role of phys has been studied, but the integration of the CRYs in the retrograde signalling pathways that tune photomorphogenesis in blue light make them interesting candidates to assess for their role in controlling dual-localized proteins that may be retrograde signals.

## Conclusions

**Fig. 1.**
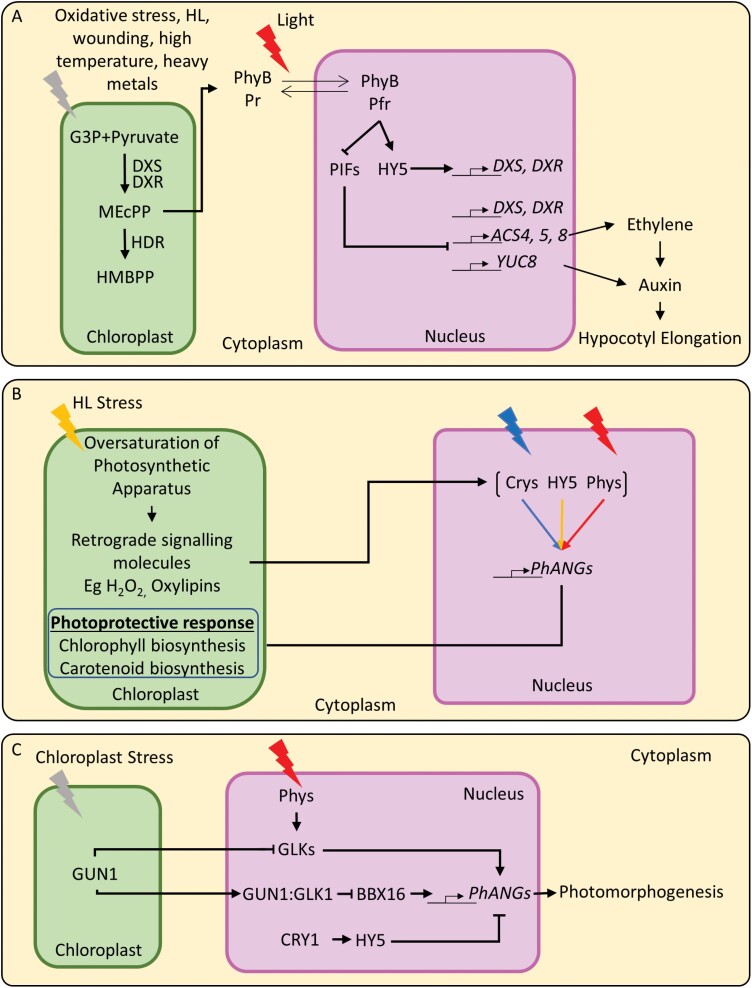
Phytochromes (phys), cryptochromes (CRYs), and HY5 integrate light and retrograde signals from the chloroplast to tune nuclear genome responses to a changing environment. (A) MEcPP tuning of phyB-modulated growth responses. Chloroplast stress-induced MEcPP accumulation increases the abundance of phyB-Pr protein. Red light-activated phyB-Pfr translocates to the nucleus to inhibit PIF activity, and target hormonal pathways to halt hypocotyl elongation. In addition to inhibiting PIF activity, phyB promotes HY5 accumulation. In a feedback loop, HY5 and PIFs antagonistically regulate the transcriptional accumulation of *DXS* and *DXR*, two of the rate-limiting steps in the MEP pathway from which MEcPP derives. (B) High light- (HL) induced stress responses are dependent on photoreceptor and HY5 activity. HL stress induces damage to the photosynthetic apparatus, triggering the release of retrograde signalling molecules including H_2_O_2_ and oxylipins, which target the phy-, CRY-, and HY5-dependent activation of *PhANG* expression and photoprotective responses including chlorophyll and carotenoid biosynthesis. (C) A GUN1-dependent pathway inhibits *PhANG* accumulation to halt photomorphogenesis in response to chloroplast stress. GUN1 antagonistically inhibits phy-mediated photomorphogenesis through a GUN1:GLK1 complex that down-regulates BBX16-mediated *PhANG* expression. CRY1 and HY5 also co-target GUN1-dependent *PhANG* accumulation in a converging pathway, contributing to the responsiveness of *PhANG*s to chloroplast stress.

The research highlighted in this review supports an emerging view that the phyand CRY photoreceptor signalling, including through transcription factors such as PIFs and HY5, intertwine with both the anterograde and retrograde signalling pathways. This crosstalk is essential for the tuning of the nuclear and plastid genomes in response to environmental cues (Fig. 1).

As part of the anterograde signalling cascades, the photoreceptors and their signalling components contribute to both nuclear and plastid transcription, post-transcription, and translational mechanisms. On the other hand, in retrograde signalling, they are not only contributors to the activation of pathways involved in the emission of retrograde signals, such as the tetrapyrrole and MEcPP pathways, but are also targets themselves of the retrograde signals (Fig. 1A). These dual functionalities are probably part of their extended capacity to optimize plant growth in response to environmental cues. In particular, *phyA* and *HY5* transcript accumulation and phyB protein abundance increased in response to retrograde signal activators such as norflurazon and the MEcPP pathway. Additionally, GUN1 signalling tunes *CRY1* and *HY5* transcript abundance and intersects with the photoreceptors in the control of de-etiolation responses. However, at present, the full reach of these cross-regulations remains to be explored, although the identification of *cry1* as a *gun* mutant hints at a wide involvement of CRYs in plastid to nucleus signalling (Fig. 1C).

CRYs, phys, and HY5 are also part of the chloroplast responsiveness to environmental cues, including the set up and the control of photoprotective mechanisms against the detrimental effects of HL. HL is emerging as a condition where the crosstalk between photoreceptors and retrograde signals is essential to optimize chloroplast functions, including the management of stress (Fig. 1C). Additionally, as part of the perception of light quality, phys, PIFs, and HY5 participate in the modulation of the shade avoidance syndrome elongation responses that are tuned via retrograde signals to the status of the chloroplast.

Finally, dual-localized proteins with capacity to act as retrograde signals, such as WHY1 and HMR, are also light quality responsive, but the impact of the phys and CRYs on their regulation is just starting to emerge.

## Abbreviations

CRY: cryptochrome
DXR: DXP REDUCTOISOMERASE
DXS: DXP SYNTHASE
ELIP2: EARLY LIGHT INDUCED PROTEIN
FC1: FERROCHELATASE 1
GUN: GENOMES UNCOUPLED
GLK: GOLDEN2-LIKE protein
HL: high light
HMR: pTAC12/HEMERA
LHCB: light-harvesting complex B
HY5: LONG HYPOCOTYL 5
MEcPP: methylerythritol cyclodiphosphate
MEP: methylerythritol phosphate
PPR: pentratricopeptide domain-containing
*PhAPG*: photosynthesis-associated plastome gene
*PhANG*: photosynthesis-associated nuclear gene
PIF: phytochrome-interacting factor
phy: phytochrome
pTAC: PLASMID TRANSCRIPTIONALLY ACTIVE CHROMOSOME
PEP: PLASTID-ENCODED POLYMERASE
ROS: reactive oxygen species
RBCS-1A: ribulose biphosphate carboxylase small subunit
TPR: tetratricopeptide domain-containing
WHY1: WHIRLY1.
